# Hématome rétro-péritonéal révélant une tumeur de la veine cave inférieure: à propos d'un cas

**DOI:** 10.11604/pamj.2016.24.93.6241

**Published:** 2016-05-27

**Authors:** Karim Lakhdar, Sidi Mohamed Malki, Ihssane Er-raji, Ali Derkaoui, Abdelkrim Shimi, Mohamed Khatouf

**Affiliations:** 1 Service de Réanimation Polyvalente A1, CHU Hassan II, Fès, Maroc; 2 Service de Radiologie, CHU Hassan II, Fès, Maroc

**Keywords:** Tumeur, veine cave inférieure, hématome rétro-péritonéal, Tumour, inferior vena cava, retroperitoneal hematoma

## Abstract

Les tumeurs de la veine cave inférieure sont rares, représentées essentiellement par le léiomyosarcome. Elles ont une présentation clinique peu spécifique, cependant, la littérature ne rapporte pas de manifestations hémorragiques. Le diagnostic préopératoire repose sur la tomodensitométrie et l'imagerie par résonance magnétique et la confirmation est histologique. Nous rapportons un cas d'hématome rétro-péritonéal révélant une tumeur de la veine cave inférieure. Le diagnostic a été posé sur des arguments cliniques et radiologiques.

## Abstract (en)

Tumors of the inferior vena cava are rare, their most common histology is represented by leiomyosarcoma. They have few specific clinical features, however, the literature does not report hemorrhagic manifestations. Preoperative diagnosis is based on tomodensitometry and magnetic resonance imaging (MRI); it is confirmed by histology. We report a case with retroperitoneal hematoma revealing a tumor of the inferior vena cava. The diagnosis was made on the basis of clinical and radiological findings.

## Introduction

Les tumeurs malignes de la veine cave inférieure (VCI) sont très rares, cependant, elles occupent une place importante dans les tumeurs à point de départ vasculaire. Plusieurs entités anatomopathologiques ont été décrites dans la littérature, le léiomyosarcome en représente 95% des cas. Le diagnostic préopératoire repose sur la tomodensitométrie et l'imagerie par résonance magnétique abdominales. Il n'est posé que dans 10% des cas.

## Patient et observation

**Figure 1:**
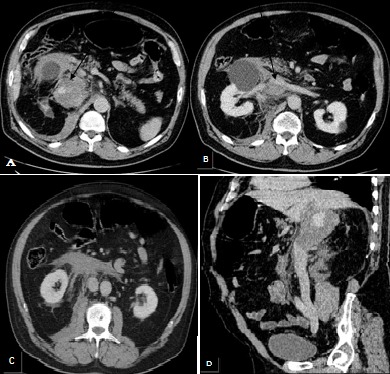
Processus tissulaire faiblement rehaussé et nécrotique rétro péritonéal droit, circonscrivant en manchon la VCI étendu depuis sa portion rétro hépatique jusqu'au pôle inférieur du rein droit (A, B, C) avec importante infiltration tissulaire de la graisse rétro péritonéale de voisinage (D)

K. M. est un patient de 66 ans, suivi pour arythmie complète par fibrillation auriculaire (ACFA) depuis 16 ans sous anti-vitamine K, admis aux urgences dans un tableau de douleurs abdominales diffuses avec distension abdominale. L'examen clinique à l'admission a trouvé un patient conscient, pâle, tachycarde à 150bpm avec des extrémités froides et une pression artérielle à 70 / 40 mm-Hg. Le patient a bénéficié d'une mise en condition avec oxygénothérapie, Prise de deux voies veineuses périphériques avec remplissage vasculaire par 1000 mL de sérum salé 0,9% et sondage vésical. Après stabilisation de l’état hémodynamique, le patient a bénéficié d'un bilan radiologique fait d’échographie et de tomodensitométrie (TDM) abdominales objectivant un processus tissulaire faiblement rehaussé et nécrotique rétro péritonéal droit, circonscrivant en manchon la VCI, étendu depuis sa portion rétro hépatique jusqu'au pôle inférieur du rein droit avec importante infiltration tissulaire de la graisse rétro péritonéale de voisinage (Figure 1). Après antagonisation de l'anti-vitamine K, un bilan biologique a été réalisé objectivant une hémoglobine (Hb) à 8,3 g/dL, un taux de prothrombine (TP) à 50%, un International Normalized Ratio (INR) à 1,57, un taux d'urée à 0,91 g/L et une créatinine à 50 mg/L. Une demande de culots globulaires, de plasma frais congelé et de culots plaquettaires a été faite pour préparer le patient a une éventuelle intervention chirurgicale. 15 minutes après son admission, l’état neurologique du patient s'est dégradé avec agitation extrême. L’état hémodynamique s'est altéré également avec tachycardie à 170bpm, sueurs profuses et anurie. Le patient a été intubé-ventilé sur des critères hémodynamiques avec prise d'une voie veineuse centrale jugulaire interne et mise sous Noradrénaline avec augmentation progressive des doses jusqu’à 2 microgrammes/kg/min. L'indication d'un traitement chirurgical a été posé, mais le patient a rapidement installé un état de choc réfractaire avec décès du patient une demi-heure après.

## Discussion

Les tumeurs de la VCI sont très rares. Elles représentent 0,07% cas dans une série autopsique. Elles sont dominées par le léiomyosarcome qui représente 95% des tumeurs primitives de la VCI. Les 5% restant sont représentés par: le fibrosarcome et les formations kystiques (lymphangiome) ou solides (reliquats embryonnaires et ganglionnaires) [1]. La VCI se divise en 3 trois segments: segment I (la VCI sous rénale), segment II (la VCI juxta-rénale et rétro-hépatique) et le segment III (la VCI sus-hépatique jusqu’à sa terminaison dans l'oreillette droite) [2]. La répartition topographique détermine la présentation clinique et la résécabilité tumorale [3, 4]. La présentation clinique est peu spécifique: masse abdominale (48%), douleurs abdominales (66%), œdèmes des membres inférieurs (39%), syndrome de Budd-Chiari (22,2%) [5].

Chez notre patient, la symptomatologie était dominée par la douleur et la distension abdominales. Plusieurs modes de révélation ont été rapportés dans la littérature [2, 6–8] (Tableau 1). Cependant, aucune complication hémorragique n'a été rapportée. L'hémorragie chez notre patient est surement liée au traitement anti-vitamine K pris pour l'ACFA. L’échographie est un examen de débrouillage, elle montre une tumeur rétro-péritonéale, inhomogène. Elle précise sa situation dans un segment anatomique de la VCI et par rapport aux organes de voisinage [2, 7]. Le Doppler renseigne sur la perméabilité de la VCI, de la veine porte, des veines rénales et veines sus-hépatiques [2]. La TDM avec contraste permet d'affirmer la masse polylobée. Elle précise la localisation, les rapports avec les organes de voisinage définissant ainsi la possibilité d'exérèse et le degré d'obstruction de la VCI. Elle permet également d'identifier des lésions métastatiques hépatiques ou pulmonaires [2]. L'IRM précise au mieux l'origine vasculaire, les rapports avec les organes de voisinage et la perméabilité de la VCI [2]. La confirmation du diagnostic est histologique. La biopsie peut être réalisée par voie percutanée sous repérage tomodensitométrique, ou par voie endo-veineuse [2]. Chez notre patient, devant l'installation rapide d'un état de choc réfractaire, la prise en charge était axée sur l'optimisation de l’état hémodynamique. Nous ne disposons donc pas de preuve histologique.

**Tableau 1:** Les différents modes de révélation des tumeurs de la VCI rapportés dans la littérature

Auteur / Année	Nombre de cas	Sexe	Age	Mode de révélation
El Malki HO/ 2003	1	Féminin	42	Asthénie, douleurs de l'hypochondre droit, troubles digestifs
Rouas L/ 2005	1	Féminin	17	Douleurs lombaires droites, masse épigastrique indolore
Bonnet S / 2006	1	Masculin	53	Douleurs épigastriques et du flanc droit
Malajati H / 2009	3	Féminin	50	Douleur abdominale, masse de l'hypochondre droit
		Masculin	50	Doleurs abdominales, sueurs
		Masculin	72	Douleurs abdominales, perte de poids
Henriquez CR	1	Masculin	31	Œdèmes des membres inférieurs

Le traitement de ces tumeurs est chirurgical, son but est l'exérèse complète de la tumeur, la conservation du retour veineux et la prévention des récidives [2]. La difficulté de la résection de la tumeur dépend surtout de sa localisation. Les tumeurs du segment I de la VCI ne posent en général que peu de problèmes dans la mesure où le clampage complet de la VCI sous-rénal est bien toléré autorisant une exérèse tumorale complète. Les tumeurs du segment III sont rarement résécables et leur résection reste conditionnée par l'envahissement des veines sus-hépatiques. Une circulation extracorporelle est souvent nécessaire. En ce qui concerne le segment II, deux problèmes se posent: la localisation rétro-hépatique de la tumeur et l'envahissement éventuel d'une ou des deux veines rénales [9]. Chez notre patient, le traitement chirurgical n'a pas pu être réalisé vu l'installation rapide d'un état de choc hémorragique réfractaire conduisant au décès. La place de la chimiothérapie et la radiothérapie dans cette pathologie reste discutée. La chimiothérapie et la radiothérapie néo-adjuvantes pourraient trouver une place pour réduire le volume tumoral avant chirurgie, alors qu'utilisées comme thérapies adjuvantes, elles pourraient être intéressantes en cas de résection incomplète [3].

## Conclusion

Les tumeurs de la VCI sont très rares. La survie à long terme dépend du diagnostic précoce et de la performance chirurgicale. L’évolution des tumeurs de la VCI reste péjorative avec un taux de survie à 5 ans inférieur à 50% et un taux de survie à 10 ans inférieur à 30%.
